# MicroRNA target homeobox messenger RNA in HIV induced hematopoietic inhibition

**DOI:** 10.3389/fcell.2024.1382789

**Published:** 2024-04-24

**Authors:** Prasad S. Koka, Bharathi Ramdass

**Affiliations:** Biomedical Research Institute of Southern California, Oceanside, CA, United States

**Keywords:** HIV, hematopoiesis and hematological disorders, microRNA, intercellular interactions, homeobox messenger RNA, post-transcriptional regulation, signaling pathways, cell lineage-fate

## Abstract

Cytopenias are a common occurrence due to abnormal hematopoiesis persistent in patients suffering from and advancing with HIV/AIDS. In order to develop efficacious therapies against cytopenias, it is necessary to understand the mechanisms by which HIV infection affects the differentiation of hematopoietic stem-progenitor cells (HSPCs), causing hematopoietic inhibition, that leads to hematological disorders. Currently, only the antiretrovirals that are being used to treat HIV infection and indirectly lower the levels of virus replication also co-attenuate cytopenias. The evidence available suggests that this indirect efficacy may not prevail for the lifetime of the infected patients, and the acquired immunodeficiency can overtake the beneficial consequences of decreased virus replication. As cited in this article, we and our colleagues are the first to make a foray into the involvement of microRNAs and their use as potential interventional treatments for the cytopenias that occur with HIV/AIDS. Herein, we progressed further in the direction of the mechanisms of the involvement of homeobox gene regulation to cause cytopenias. We had previously shown that HIV-1 inhibits multi-lineage hematopoiesis of the CD34^+^ cells using SCID-hu Thy/Liv animals *in vivo*. Furthermore, we demonstrated that the virus-induced hematopoietic inhibition occurred despite the CD34^+^ cells being resistant to HIV-1 infection. We set out to search for the specific host factors secreted by CD4^+^ T-cells that likely participate in the inhibition of hematopoiesis of the HIV infection-resistant CD34^+^ cells. More recently, we reported the identification of virus-infected CD4^+^ thymocyte-secreted miRNA-15a and miRNA-24 and that their differential expression following HIV infection causes the indirect inhibition of hematopoiesis. We then hypothesized that the observed miRNA differential expression in the virus-infected T-cells causes the abnormal regulation of homeobox (HOX) gene-encoded transcriptomes in the CD34^+^ cells, affecting specific MAPK signaling and CD34^+^ cell fate, thereby disrupting normal hematopoiesis. We present that in HIV infection, miRNA-mediated post-transcriptional dysregulation of HOXB3 mRNA inhibits multi-lineage hematopoiesis, which translates into hematological disorders in virus-infected patients with HIV/AIDS. These observations portend specific microRNA candidates for potential efficacy against the virus-induced cytopenias that are otherwise not treatable by the existing HAART/ART regimens, which are primarily designed and applicable for the attenuation of virus replication.

## Introduction

Cytopenias are common, unrelenting, and debilitating occurrences in HIV-infected individuals (Koka and Reddy, 2004; Opie, 2012; Durandt et al., 2019). Millions of people worldwide have been infected with HIV, and a significant proportion of these infected patients develop chronic pancytopenias due to the continuous replication and emergence of new virus variants, exhausting the virus-infected patients’ ability to mount potent immune responses (Tamir et al., 2019; Getawa et al., 2021; Marchionatti and Parisi, 2021). These cytopenias are caused by the depletion or insufficiency of blood-forming cells, including T and B lymphocytes, and affect the immune responses that fight opportunistic infections. The virus invasion into humans reduces the dual role of possessing the virus’ target receptor and host immune CD4^+^ T-cell counts to <200 cells/µL, at which time these patients also begin antiretroviral drug therapy (Volberding et al., 2004; Antiretroviral Therapy Cohort Collaboration et al., 2008). Such general and overall anemic conditions are generally found in patients with advanced HIV antigen expression markers in the infected individuals. In the initial and subsequent earlier stages of human immunodeficiency virus (HIV) infection, antiretroviral treatments (ARTs) generally provide efficacy in containing cytopenias. This is primarily due to the indirect effects of these antiviral therapies, which coincidentally achieve the attenuation of viral replication and concomitantly target CD4^+^ thymocyte survival to the maximum possible levels. Approximately 10^**11**^ new erythrocytes and platelets are required to be produced daily in an average, normal individual. In individuals with advanced HIV infection and at the initiation of HAART, erythropenia (anemia), leukopenia, thrombocytopenia, *etc.*, are common because these normal levels of blood cell production are compromised due to the exhaustion of their immune systems that are being sustained by the antiretroviral treatments (Firnhaber et al., 2010; Kyeyune et al., 2014; Fiseha and Ebrahim, 2022). However, patients survive with subdued but chronic HIV-infection-mediated side effects despite the initial and periodic mitigation of the persistent immunodeficiency conditions due to antiretroviral ART/HAART intake. In both adult and pediatric patients, the decreased efficacy to maintain immune status closer to normal, eventually leads to the development of chronic cytopenias (Ambler et al., 2012; Fan et al., 2020; Madzime et al., 2021; Nuru et al., 2023; Tan et al., 2023). Thus, there is still a large gap to fill between the development of efficacious drugs against cytopenias that are independent of antiretrovirals (ART/HAART) due to the need to achieve efficacy in both the attenuation of HIV replication and containment of multiple cytopenias specific to the afflicted individuals in a patient-specific, drug-selective manner of antiretroviral therapies. Non-ART/HAART-dependent treatments without clinically serious symptomatic side effects and efficacy that can last for a lifetime for all HIV-infected patients enduring multiple cytopenias have yet been elusive to date, as inferred from the content of the above-referenced citations from various investigators.

**FIGURE 1:**
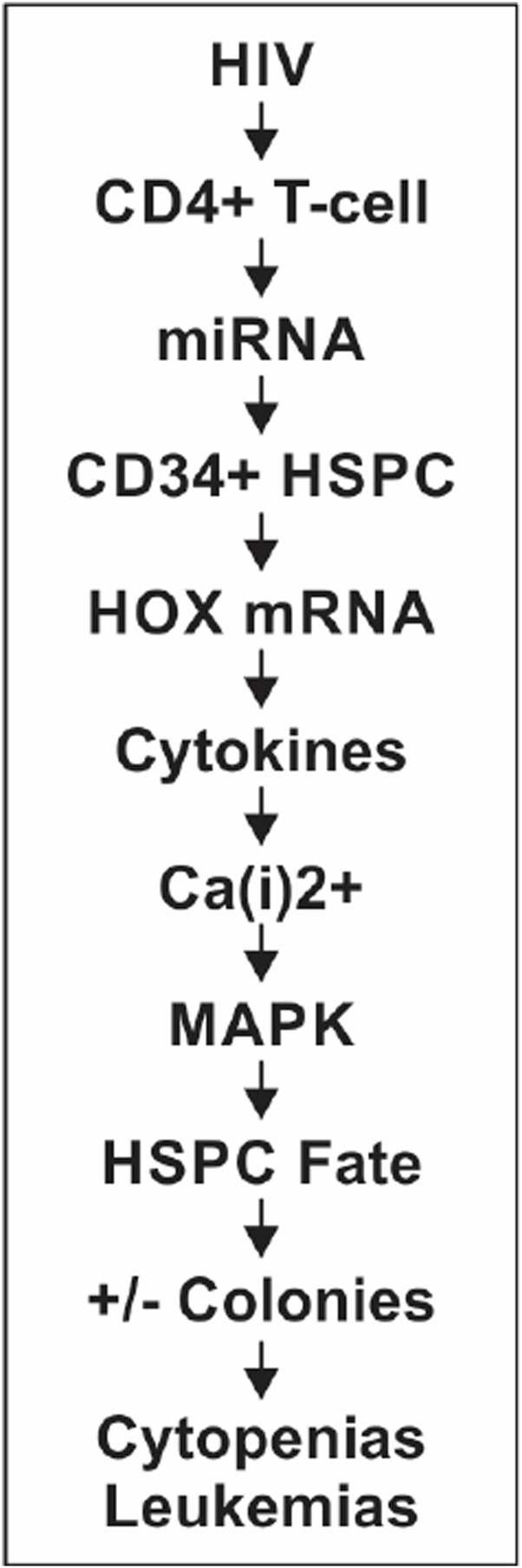
Sequence of cellular, molecular, and functional events.

In continuation of our experimental studies and investigations during the last 20+ years, we have moved closer to the goals of addressing not only the cellular and molecular mechanisms of cytopenias that afflict HIV-infected individuals but also clues and potential candidate drugs that may well be compatible biologically, as elaborated in the following sections. We postulate and hypothesize that HIV infection induces hematopoietic dysfunction via the infected CD4^**+**^ T-cell-mediated microRNAs that will target the homeobox messenger RNA (HOX mRNA) of the CD34^**+**^ hematopoietic stem-progenitor cells (HSPCs) present in the cellular microenvironment post-transcriptionally, consequently affecting these cells’ fate to cause hematopoietic inhibition (Figure 1). The chain of events leading to abnormal hematopoiesis and resulting in the depletion or loss of blood cell formation in HIV-infected patients is outlined concisely (Figure 1), and the mechanistic details and experimental evidence are presented and narrated in detail.

### HIV-1 inhibition of hematopoiesis *in vivo* is an indirect effect stemming from the primary target cells

In order to develop efficacious therapies against specific cytopenias, it is necessary to understand the mechanisms by which HIV infection affects the differentiation of HSPCs, causing hematopoietic inhibition vis-à-vis the subsequent hematological disorders (cytopenias) (Figure 1). In this regard, we had previously reported that HIV-1 inhibits the differentiation of HSPCs when the CD34^+^CD38^−^ cells that were derived from the virus-infected human fetal thymus + liver tissues engrafted severe combined immune deficiency mice (SCID-hu Thy/Liv) *in vivo* (Koka et al., 1998). These cells were analyzed *ex vivo* for their potential to form mature, terminally differentiated lineage-specific colonies in a semi-solid methylcellulose medium enriched with the required growth factors or cytokines (STEMCELL Technologies, Vancouver, BC, Canada) that support the erythroid BFU-E and myeloid CFU-GM lineage colony formation (Koka et al., 1998). Subsequently, we also reported that this hematopoietic inhibition of CD34^+^CD38^−^ cells that were yet to acquire the mature lineage committed markers was an indirect phenomenon or event and that these HSPCs were HIV infection-resistant (Koka et al., 1999).

### Resurgence of virus-inhibited hematopoiesis in a fresh stromal microenvironment *in vivo*

Furthermore, we showed that the virus-exposed CD34^+^ cells that exhibited a decreased hematopoiesis when derived and analyzed *ex vivo*, upon 3 weeks post-re-engraftment into the secondary (SCID-hu) Thy/Liv animal stromal microenvironment *in vivo*, showed a partial hematopoietic resurgence/recovery when derived and reanalyzed *ex vivo* (Koka et al., 2004). Thus, our findings suggested that the intercellular interactions between the CD4^+^ T-cells and CD34^+^ hematopoietic progenitor cells that prevailed in the primary SCID-hu Thy/Liv implants could be the responsible factors for the inhibition of hematopoiesis. This further suggests that the putative host miRNA cellular factors, as described in the following section, segregated with the CD4^+^ cells of the infected primary animal graft and were lacking (un-induced) in the new stromal microenvironment of the secondary animal graft comprising the fresh CD34^+^ cells, thus preventing a repeat deleterious influence anew. Furthermore, favorable conditions such as the absence of HIV-1 infection in the secondary recipient animals and, consequently, the purported absence of miRNA-dysregulated thymocytes helped in hematopoietic recovery. This strengthened our hypothesis that host cellular factors, most likely generated from the virus-infected CD4^+^ thymocytes, were indirectly and negatively influencing the CD34^+^ cells present within the primary stromal cellular microenvironment in SCID-hu Thy/Liv *in vivo*; however, in contrast to that, virus-induced pathogenesis did not exist in, nor was it carried forward into, the stroma of the secondary recipient animal grafts.

### Identification of the miRNA host factors involved in HIV-1-induced hematopoietic inhibition

In continuation of our *in vivo* experimental studies and reports as stated above, we embarked on the identification of the host cellular factors involved in the HIV-induced hematopoietic inhibition through “narrower” model studies, viz., *in vitro,* to facilitate the determination of the nature of the HIV/CD4/CD34 interactions. We envisaged and set out to isolate the putative cellular factors, presumed to be small molecular in nature, using cut-off filters that harvested concomitant cell-free and virus-free supernatants (Padmanabhan et al., 2020). These supernatants inhibited hematopoietic colony formation *in vitro*. We then demonstrated that in HIV-1 infection, the inhibition of CD34^+^ HSPC differentiation occurs due to a dynamic differential regulation of two microRNAs identified thus far, miR-15a (downregulated) and miR-24 (upregulated), which are secreted by the virus-infected CD4^+^ T-lymphocytes and indirectly target(ed) the CD34^+^ cells to inhibit hematopoiesis (Padmanabhan et al., 2020).

### Functions of miR-15a and miR-24 in benign and malignant hematopoiesis, as reported by other investigators

In humans, miR-15a is found at the chromosome locus 13q14 (Calin et al., 2002; Pekarsky et al., 2018) and shares a homology with messenger RNA of the *Bcl-2* gene (Josefsen et al., 2000)**.** MicroRNA-15a is commonly deleted or downregulated in chronic lymphocytic leukemia and controls the expression of the *Bcl-2* gene (Pekarsky et al., 2018). Interestingly, Bcl-**x**_**L**_, a homolog of Bcl-2, is upregulated during CD34^+^ cell differentiation into erythrocytes but not during CD34^+^ cell differentiation into granulocytes (Josefsen et al., 2000)**.** MicroRNA-24 has been reported to modulate erythropoiesis, is located along with miR-23 and miR-27 on human chromosomes 9 and 19, and is conserved in various species (Lal et al., 2008). In primary human CD34^+^ hematopoietic progenitor cells, miR-24 levels negatively correlate with activin levels. Overexpression of miR-24 impairs erythropoiesis by downregulating activin and inhibiting miR-24 accumulation. MiR-24 increase otherwise represses the activin-mediated accumulation of hemoglobin, an erythroid differentiation marker (Shenoy and Blelloch, 2014). MicroRNA-24 has also been shown to suppress the expression of two crucial cell-cycle control genes, E2F2 and Myc, in hematopoietic differentiation (Lal et al., 2009). MicroRNA-24 enhances myeloid proliferation but blocks differentiation by directly downregulating MAPK phosphatase-7 and activating JNK and p38 kinases (Zaidi et al., 2009). Conversely, it was reported (Roy et al., 2015) that miR-24 is required for differentiation in the development of HSPCs. The inhibition of miR-24 in embryonic stem cells (ESC) does not affect the generation of blast colony-forming cells (BL-CFCs) but does compromise the ability of those BL-CFCs to produce HSPCs through downregulation in the expression of Scl and Runx1.

Runx1 is a transcription factor that is on chromosome 21, and Down’s syndrome patients show an increased dosage of Runx1 (trisomy 21) and have an increased risk of developing acute megakaryoblastic leukemia (Izraeli et al., 2007). When Runx1 localization is altered, it causes an increase in miR-24. These results identify miR-24 as a requirement for erythrocyte differentiation and the formation of erythrocytes (Roy et al., 2015).

### MicroRNA intrinsic to and associated with CD34^+^ hematopoietic stem-progenitor cells and CD4^+^ thymocytes

MicroRNAs associated with and intrinsic to CD34^+^ cells are involved in the functions that regulate hematopoiesis (Choong et al., 2007; Georgantas et al., 2007; Bhagavathi and Czader, 2010; Petriv et al., 2010; Lazare et al., 2014). HIV-1-receptor CD4^+^ T-cells also express miRNAs (Rossi et al., 2011; Torri et al., 2017). However, since the HIV-1-induced hematopoietic inhibition that is influenced by the CD4^+^ T-cells is indirect, the CD34^+^ HSPC-intrinsic-miRNAs may not be perturbed in order to self-regulate CD34^+^ intracellular functions that determine their own cell-fate, until the “arrival” of the abnormally modulated miR-15a and miR-24 at the site of CD34^+^ cells, following HIV infection. Earlier reports of the association of miRNAs expressed by either CD34^+^ HSPC or CD4^+^ T-helper cells were not known to be dependent on any role or influence of HIV-1 infection in the context of dys/regulation of hematopoiesis. There have been reports of the role of miRNAs in CD4^+^ T cells post-HIV infection, as in blocking HIV infection (Chiang and Rice, 2012), T-cell activation following HIV infection (Zhao et al., 2017), and modulating cytokine secretion from CD4^+^ T-cells (Swaminathan et al., 2012). HIV infection reportedly causes changes in the miRNA profile of CD4^+^ T-cells (Bignami et al., 2012), which supports our *hypothesis* in this article.

### Influence of HIV-1 infection on CD4^+^ thymocyte-released miRNA-mediated indirect hematopoietic inhibition

MicroRNAs have been previously implicated in the post-transcriptional regulation of HOX transcription factor genes through the destabilization of the HOX messenger RNA (Yekta et al., 2004; Garzon et al., 2008) (Table 1; Table 2) (Shimamoto et al., 1998; Björnsson et al., 2001; Björnsson et al., 2003; Brun et al., 2003; Sevinsky et al., 2004; Argiropoulos and Humphries, 2007; Geest and Coffer, 2009; Alharbi et al., 2013). We reported (Padmanabhan et al., 2020) that the two human T-cell-specific miRNAs, miR-15a (Cimmino et al., 2005; Zhao et al., 2009) and miR-24 (Wang et al., 2008; Roy et al., 2015), were differentially regulated in HIV-1 infection and impaired myelopoiesis (CFU-GM) and erythropoiesis (BFU-E) of the CD34^+^ cells**.** Our data showing HIV-1-mediated decrease in the CD34^+^ cell colony-forming activity (CFA) in the context of concomitant miR-15a downregulation and miR-24 upregulation suggest potential mechanistic implications for virus-induced hematopoietic inhibition, hence, the onset of hematological disorders in the virus-infected patients (Table 2). This is the first report of an HIV-1-mediated, presumably dynamic, differential regulation of miR-15a and miR-24 by the virus-infected CD4^+^ T-cells to induce the inhibition of hematopoiesis, viz., indirectly inhibit CD34^+^ cell differentiation.

**TABLE 1:** Homeobox gene regulation of signaling pathways and the influence on the differentiation of lineage-specific HSPCs. The dual up–down (blue-dotted-black) arrows for HOXB3-driven CFU-GM potential denote the hematopoiesis of progenitors and decrease in proliferation, respectively. The dual down–up (blue–red) arrows for CFA due to HOXA10 enhancement denote decrease in progenitors and increase toward malignant hematopoiesis, respectively. References:Shimamoto et al., 1998; Björnsson et al., 2001; Björnsson et al., 2003; Brun et al., 2003; Sevinsky et al., 2004; Yekta et al., 2004; Argiropoulos and Humphries, 2007; Geest and Coffer, 2009.

Homeobox transcriptome expression	Signaling pathway		Colony-forming activity		
	MAPK	ERK	BFU-E	CFU-GM	CFU-MK
HOXB3**↑**	**↑**	**↑**	**↑**	**↑** 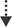	**↑**
HOXB4**↑**	**↑**	**↑**	**↑**	**↑**	**↑**
HOXA10**↑**	**↑**	**↑**	**↓↑**	**↑**	**↓↑**

**TABLE 2:** Consequences of HIV-1 infection in the context of reported miRNA-mediated post-transcriptional regulation of HOX mRNA and HSPC fate/loss, as assessed by CFA. References: Lawrence et al., 1996; Magli et al., 1997; Fox et al., 2003; Yekta et al., 2004; Alcalay et al., 2005; Garzon et al., 2008; Shah et al., 2011; Kohanbash and Okada, 2012; Padmanabhan et al., 2020.

HIV-1		MicroRNA		Homeobox mRNA			Colony-forming activity		
CXCR4**+**	CCR5**+**	miR-15a**↓**	miR-24**↑**	HOXB3**↓**	HOXB4**↓**	HOXA10**↑**	BFU-E**↓**	CFU-GM**↓**	CFU-MK**↓**

### Mechanisms of growth factor regulation impact HIV-1-induced hematopoietic inhibition

These mechanisms are expected to include the miRNA-mediated dys/regulation of cytokine genes (Ward et al., 2000; Asirvatham et al., 2009), such as those of IL-3, IL-6, GM-CSF, and SCF, or different poietins, such as promegapoietin-1a (PMG-1a), angiopoietins-1/2 (Ang-1 and Ang-2), and the multi-lineage (but primarily megakaryocyte)-influencing ligand thrombopoietin (TPO) (Kohanbash and Okada, 2012; Haetscher et al., 2015). TPO binds to its natural receptor, c-Mpl, encoded by the cellular *proto-oncogene* of myeloproliferative leukemia, *c-mpl* (the viral counterpart is *v-mpl*), which is expressed on the CD34^+^ HSPCs to primarily regulate megakaryopoiesis but also has relevance in regulating differentiation into other lineages (Koka et al., 2004; Sundell and Koka, 2006; Zhang et al., 2009; Zhang et al., 2010). Together, these growth factors and poietins influence the fate of CD34^+^ cells, viz., multi-lineage formation by acting on HOX gene regulation, as assessed by CFA (Sauvageau et al., 1994; Lawrence et al., 2005; Auvray et al., 2012; Liu et al., 2013). The small, mature non-coding RNAs, typically ∼19–25 nucleotides (nt) in length (miRNA <26 nt), derived from long non-coding RNAs (lncRNA, >200 nt), including those implicated above (miR-15a and miR-24), target specific messenger RNAs (mRNA), viz. those of the relevant homeobox (HOX) genes; this targeting causes abnormal transcriptome (transcription factor) protein expression, possibly leading to protein degradation. Hence, such miRNA-mediated mechanisms of regulation that primarily pertain to instances of expression of the growth factor cytokines that, in turn, influence the lineage commitment and differentiation of hematopoietic progenitor cells. These regulatory mechanisms will be expected to occur via the post-transcriptional regulation of homeobox (HOX) genes, which encode transcription factors generally intrinsic to HSPCs (Sauvageau et al., 1994; Lawrence et al., 1996; Magli et al., 1997; Alcalay et al., 2005; Lawrence et al., 2005; Auvray et al., 2012). Thus, the dysregulation of HOX transcription factor expression can affect both the lineage commitment of the pluripotent or multipotent progenitor cells and also the downstream differentiation of the committed progenitors into terminally differentiated lineage cells in an uncontrolled manner. These changes, in turn, dysregulate CD34^+^ cell differentiation and also affect the transcriptional regulation of growth factors, such as *TGFβ2*, which is involved in the proliferation of lineage-committed myeloid progenitors in hematopoietic malignancies such as leukemia (Shah et al., 2011). Certain other cytokine signals involved in the lineage commitment and differentiation of the common pluripotent hemangioblast precursors of the dual downstream differentiated multipotent CD133^+^ endothelial and CD34^+^ hematopoietic progenitors are also influenced (Fox et al., 2003).

## Changes in specific HOX mRNA expression levels due to post-transcriptional dysregulation by miRNAs: implications on HIV-induced cytopenias

**FIGURE 2:**
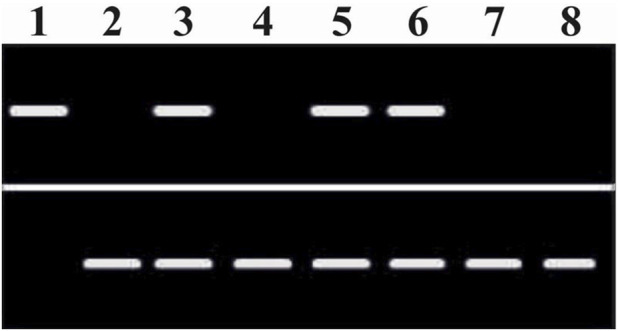
Modulation of homeobox (HOXB3) messenger RNA expression in the CD34^+^ HSPCs by the putative microRNA (miR-15a and miR-24) differential regulation and following their secretion into the supernatants (SUP) upon HIV-1 infection of the CD4^+^ T-cells. Top bands: HOXB3. Lane 1: HOXB3 (+ve control). Lane 2: GAPDH (+ve control). Lane 3: CD34+MEDIUM. Lane 4: CD4+MEDIUM. Lane 5: CD34+HIVSUP (w/o CD4^+^ cells). Lane 6: CD34+UNINF.CD4SUP. Lane 7: CD34+HIVINF.CD4SUP. Lane 8: CD4+HIVINF.CD4SUP. Bottom bands: GAPDH. RT-PCR analyses were performed with appropriate primers as applicable for the cDNA synthesis and/or subsequent use of the forward and reverse primer pairs for the relevant mRNA detection and analyses (OriGene Technologies Inc., Rockville, MD, USA). Consequential inhibitory effects on hematopoiesis: Total multi-lineage colonies (BFU-E + CFU-GM + CFU-MK) formed by the intact viable CD34^**+**^ cells, lane-wise CD34^**+**^ cell colony-forming activity (normalized %CFA), from lanes 3–8, approximated to the nearest 10: 100; 0; 90; 90; 10; and 0. Thus, the HOXB3 mRNA expression is necessary for the hematopoietic colony formation.

**FIGURE 3:**
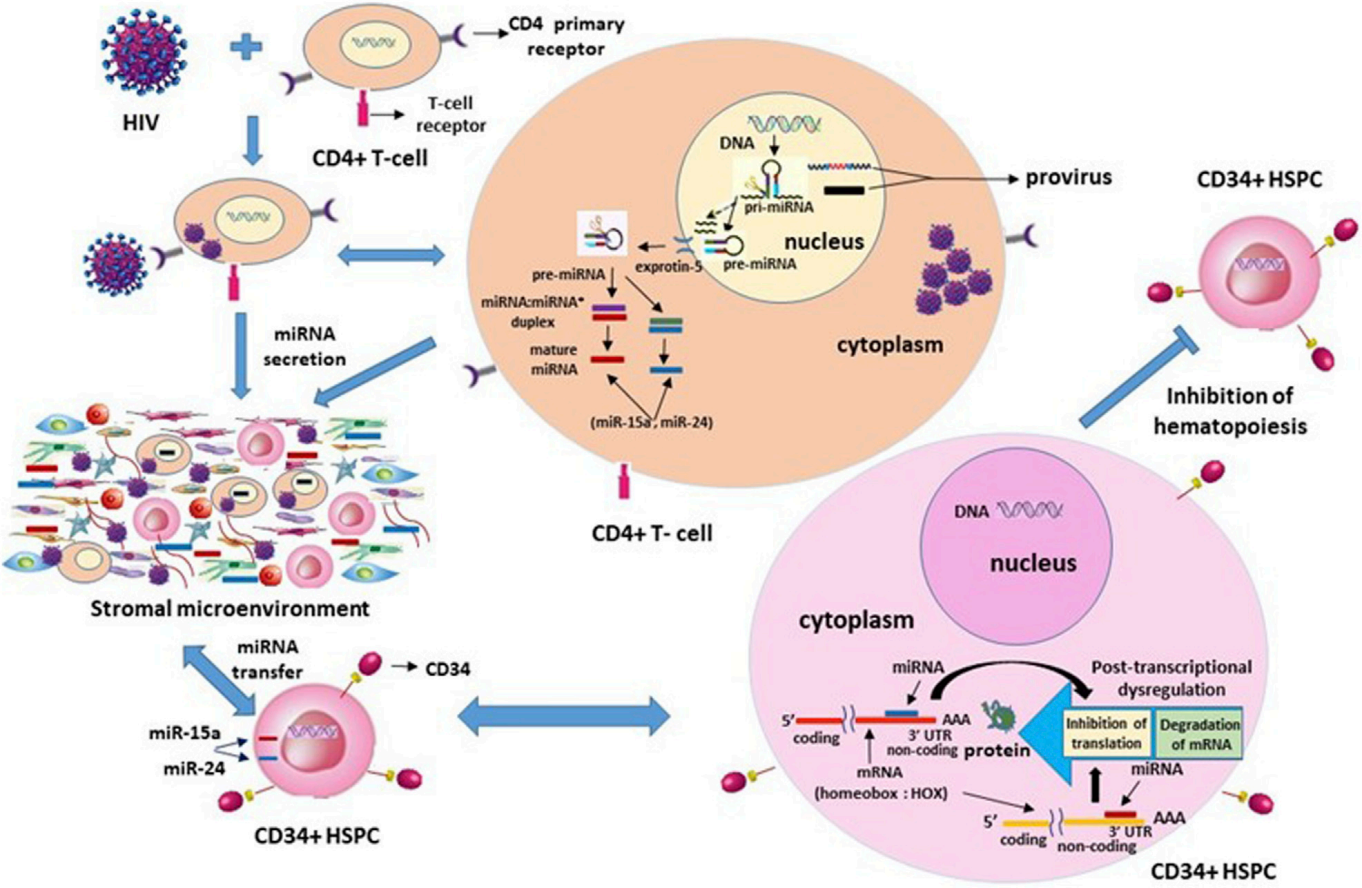
Projected mechanism of intercellular post-transcriptional (dys)regulation of homeobox messenger RNA (HOX mRNA) of the CD34^**+**^ hematopoietic stem-progenitor cells by differentially regulated microRNAs, miR-15a and miR-24, secreted into the stromal cellular microenvironment by CD4^**+**^ thymocytes upon HIV-1 infection. NOTE: In the CD4^**+**^ T-cell, the two miRNAs, miR-15a and miR-24, with multiple colors are, as depicted, combined, or encompassed into a single structure of pri-miRNA or pre-miRNAs.

The link between both the specific cytopenias and (or, vis-à-vis) HOX genes due to the consequences of HIV infection on miRNA dysregulation is necessary to be understood to advance the mechanistic and therapeutic studies. The effects of these (miR-15a and miR-24) and other miRNAs on the hitherto unreported *HOX* gene association in the context of HIV for the identification of the interactions between the miRNAs and putative target HOX transcripts are discussed herein, thus making it the first such HIV–miRNA–mRNA(HOX) association to be presented. Both HOXB4 and HOXC4 reportedly expanded the immature CD34^+^ HSPCs by 3- to 6-fold *ex vivo* (Auvray et al., 2012). However, the attribution of the lineage commitment specificity function of/to the HOX proteins is an indicator of hematopoietic cell fate. Hence, HIV intervention in the normal biological process of stem cell fate is to be reckoned with in any miRNA drug development to address the virus-induced cytopenias. Specific HOX genes have been reportedly implicated in the regulation of hematopoiesis, with miRNAs regulating HOX transcription factor gene expression at post-transcriptional levels (Choong et al., 2007; Georgantas et al., 2007; Bhagavathi and Czader, 2010; Petriv et al., 2010; Lazare et al., 2014). We hypothesize that HIV-1-induced hematopoietic inhibition, which we characterized to be via miRNAs, targets the HOX transcripts and alters the levels of specific HOX-encoded transcription factor proteins that regulate hematopoiesis [Table 1 and Table 2: REFS: (Shivdasani, 2006; Havelange and Garzon, 2010; Undi et al., 2013); Figure 2; Figure 3], causing cytopenias in HIV-infected patients.

### Effects of HIV-1 infection on CD34^+^ hematopoietic stem-progenitor cell differentiation and lineage cell fate

Specific miRNAs regulate HOX mRNAs post-transcriptionally, causing changes in the signaling pathways, which then alter and determine the hematopoietic cell fate (Sauvageau et al., 1994; Lawrence et al., 2005; Georgantas et al., 2007; Auvray et al., 2012; Barbu et al., 2020). These CD34^+^ HSPCs’ fate changes result in altered terminally differentiated lineages of the multipotent HSPCs. Based on our published results (Padmanabhan et al., 2020), we hypothesize that upon HIV-1 infection of the CD4^+^ thymocytes, the observed differential regulation of miR-15a and miR-24 that occurs causes abnormal post-transcriptional regulation of specific HOX gene-encoded transcriptomes (Koka and Ramdass, 2023). Loss or abnormal translational entities, in turn, affect specific MAPK signaling and CD34^+^ cell fate, causing the dysregulation of hematopoiesis. The mitogen-activated protein kinase kinase-1/2 (MEK1/2)/extracellular signal-regulated kinase-1/2 (ERK1/2) signaling pathways promote the differentiation of CD34^+^ HSPCs, also suggesting the involvement of intracellular Ca^2+^ [Ca(i)^2+^] signaling (Harada et al., 2014; Chakraborty et al., 2016) in HIV-1-induced hematopoietic inhibition, as shown in Figure 1. Furthermore, from this figure, the implications of HOX gene expression changes (HOXB3 and HOXA10; Table 1) in both inhibition of CD34^+^ HSPC differentiation contributing to cytopenias and the potential uncontrolled proliferation of these progenitor stem cells to cause malignancies are evident. Of particular interest may be the dual contrasting roles of specific HOX gene expression changes in infectious (HIV/AIDS) and non-communicable—unless certain virally induced—malignant (leukemia) hematological disorders or diseases.

### Signaling pathway involvement following miRNA-CD34^+^ cell interactions that affect normal hematopoietic cell fate

**FIGURE 4:**
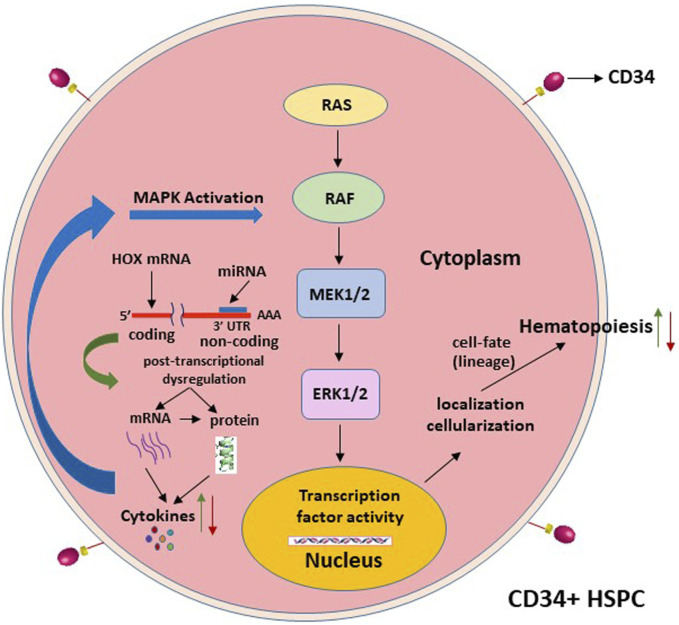
Projected signaling pathway mechanisms that alter the hematopoietic stem cell-lineage-fate, following the post-transcriptional dysregulation of the homeobox expression, as depicted in Figure 3.

The changes in specific signaling pathways that are intrinsic to the functions of HOX-mediated regulation of hematopoiesis ultimately decide the hematopoietic cell fate and, hence, alter the patterns and mechanisms of CD34^+^ cell differentiation. MicroRNA-15a has been implicated in the regulation of mitogen-activated protein kinase (MAPK) pathways, which include the mitogen-activated protein kinase kinase-1/2 (MEK1/2)/extracellular signal-regulated kinase-1/2 (ERK1/2) signaling pathway, and plays a significant role in cell survival and cell fate (Chakraborty et al., 2016). These pathways are activated by growth factor cytokines such as IL-3 and GM-CSF (even possibly by the soluble factor sFlt-1) in the hematopoietic progenitor stem cells, suggesting the involvement of intracellular Ca^2+^ [Ca(i)^2+^] signaling (Harada et al., 2014). Inhibitors of Ca(i)^2+^ signaling decrease HSPC differentiation. HOXC6 promotes cell proliferation via the activation of the MAPK pathway (Yang et al., 2019). Other HOX genes, such as *HOXD4*, are also implicated in MAPK signaling pathway alterations and affect benign or malignant hematopoiesis (Shenoy et al., 2022). MicroRNAs play a major epigenetic role in the regulation of hematopoiesis via different signaling pathways (Shen et al., 2008; Yao et al., 2019). Thus, the chain of events from altered miRNA regulation of CD34^+^ cell differentiation with the express involvement of *HOX* gene-regulated signaling pathways will address the mechanisms for the development of miRNA drug therapies for HIV-1-induced hematopoietic dysfunction (Figure 4).

### Potential cellular-pathogen biology in microenvironment interactions dependent on the multi-functional roles of miRNA

Although HIV-1 in our studies is seemingly promoting an abnormal miRNA-mediated dysregulation of hematopoiesis (Padmanabhan et al., 2020), reports also suggest that miR-24 might play a role in mitigating the challenges from other respiratory and influenza viruses by targeting the MAPK signaling pathways (McCaskill et al., 2017), potentially exhibiting multi-functional roles that may or may not be inadvertent but possibly due to the influence of different pathogens. It is certainly not uncommon for host defense mechanisms to come to the fore when facing external threats from pathogens. The implications of such observations (Lecellier et al., 2005; Egana-Gorrono et al., 2014; Reynoso et al., 2014) are clear and support our *hypothesis* and the need to thoroughly investigate and further understand the mechanisms of HIV-1-induced hematopoietic inhibition. It is also possible that between miR-15a and miR-24, one may be playing an adversarial and the other a defensive role for either the human host or the virus pathogen in our observed differential regulation of hematopoiesis. MicroRNA signatures were reported in cardiac intracellular Ca^2+^ [Ca(i)^2+^] signaling, in which miR-24’s role and MAPK/ERK1/2 were suggested (Harada et al., 2014), which also affect cell fate, as previously stated (Chakraborty et al., 2016). Hence, it is necessary to determine how these two and other control miRNAs (Choong et al., 2007; Georgantas et al., 2007; Bhagavathi and Czader, 2010; Petriv et al., 2010; Rossi et al., 2011; Lazare et al., 2014; Torri et al., 2017) target HOX mRNAs and signal the MAPK pathways.

### Understanding the mechanisms of hematopoietic dysfunction in this viral infection is key to the development of interventional therapies using microRNAs as drug therapeutic candidates for the treatment of hematological disorders in HIV/AIDS or leukemia

The HOX gene-encoded transcription factor proteins are implicated in/for the conferment of lineage specificity/commitment of the primitive hematopoietic cells for subsequent differentiation. Previously, it was reported by other investigators that when they examined A and B clusters of 16 different homeobox genes, they found that *HOXB3* and *HOXA10* were differentially regulated in the most primitive CD34^+^ cell subsets compared to their control subpopulations that lacked CD34 expression in these cell subsets (Sauvageau et al., 1994). All these cells were derived from the human bone marrow of normal individuals. Furthermore, they observed that the expression of the *HOX* genes segregated with yet-to-acquire lineage marker-negative primitive CD34^+^ cell subsets compared to the more mature lineage-positive cells within the bone marrow-derived cell subpopulations. As shown in Table 2 **,** there exists a modulation of the expression of miRNA vis-à-vis HOX-specific changes that may well determine the lineage cell fate. Therapeutic reversal of adverse cell fate changes could well be addressed by exogenous treatments with the relevant miRNAs that are characterized for their involvement in these episodes of HIV-induced cytopenias, or in leukemias, including those in other such virus-activated incidences.

### Potential need to regulate miRNA-mediated HOX gene expression to balance between the occurrence of cell depletion that causes HIV-induced cytopenias and inadvertent uncontrolled cell proliferation that can cause leukemia

The potential involvement of HOX genes in both cytopenias and leukemias require the need to regulate HOX expression of the CD34^+^ HSPCs so that the balance is maintained between these two pathogenic conditions and either disease extremities are not exacerbated. HIV-induced under-expression or downregulation of miRNAs is easier to overcome through exogenous administration than virus-induced over-expression or upregulation of miRNAs, which will require scavenging this extra substance from the systemic circulation *in vivo*. Furthermore, the involved and required miRNA levels for homeostasis will need to be carefully evaluated and established in preclinical models and through clinical trials.

### Need of the hour—transcriptomics to identify miRNA target HOX mRNA and proteomics to analyze its translational impairment

Identification of the specific miRNA-targeted putative HOX mRNA transcriptomes using RNA-sequence analysis will reveal the mechanisms of post-transcriptional dysregulation and changes in the expression levels of HOX proteins that can cause a chain of molecular events and, thus, depletion of the blood-forming cells. Shortages of these terminally differentiated cells from their precursor CD34^+^ HSPCs hamper the normal levels of immune response cells or include the abnormal differentiation into T/B-lymphocytes and render HIV-infected patients susceptible to various opportunistic infections. Thus, this will compound the immune system’s fight against virus containment and further exacerbate the health conditions of the already HIV-induced immunodeficiency-experiencing individuals. Heatmaps and volcano plots derived from the virus-infected samples need to be generated to analyze the specific HOX mRNA transcription patterns of CD34^+^ HSPCs that are exposed to the specific miRNA molecular entities released by the neighboring HIV-infected CD4^+^ T-lymphocytes. The otherwise virus-induced disproportions of the miRNA molecules need to be exogenously corrected in their levels to contain cytopenias, should the antiretroviral treatments be given up at some point during and despite their efficaciousness on cytopenias conferred in parallel by the ART drugs-induced attenuation of the HIV replicative capacity.

## Summary and conclusion

### The continuing saga of HIV induced cytopenias—do antiretrovirals suffice as/for therapies—the yet elusive need for these specific systemic conditions-needed alternatives to ARTs

We showed that HIV-1 inhibits the differentiation of human CD34^+^ cells (Koka et al., 1998), and this virus-induced hematopoietic inhibition occurs independent of and in the absence of productive virus infection of the CD34^+^ cells, which we have shown to be resistant to HIV-1 infection (Koka et al., 1999). We also showed that when the virus-exposed infection-resistant CD34^+^ cells *in vivo* were derived *ex vivo* and re-engrafted into a fresh secondary stromal microenvironment *in vivo*, in the absence of the virus’ influence, recovery and resurgence of hematopoiesis would occur (Koka et al., 2004). Hence, we hypothesized and tried to identify the human host CD4^+^ T-cellular factors that segregated with the virus’ influence on the infected CD4^+^ thymocytes that play the molecular mechanistic role in this virus-induced indirect inhibition of hematopoiesis. We have now established that HIV-1-infected human host CD4^+^ thymocytes secrete and differentially regulate the host miRNA factors, miR-15a and miR-24, to cause inhibition of differentiation of these virus infection-resistant CD34^+^ cells in an indirect manner. Thus, our observations were the first reported findings linking HIV-1 infection to dynamic differential regulation of specific miRNAs, which in turn inhibit hematopoiesis to cause virus-induced hematological disorders in infected patients with HIV/AIDS (Padmanabhan et al., 2020). Homeobox gene-encoded transcriptomes are expected to play an important role in the onset of cytopenias (Koka and Ramdass, 2023). Furthermore, herein we show that (Figure 2) HIV-1 infection caused changes in microRNA that disrupt homeobox HOXB3 *versus* control GAPDH messenger RNA (Zhou et al., 2001)**,** with the consequences of inhibiting hematopoiesis due to the loss of HOXB3 messenger RNA expression (Figure 2) (Sauvageau et al., 1997).

MicroRNAs have distinct cell-type-specific expression patterns but also exhibit “phenotypic miRNA” patterns that are specific to the developmental stage. miR-15a and miR-24 are expressed by CD4^+^ T-cells, but hematopoiesis occurs in CD34^+^ cells. During normal hematopoiesis in CD34^+^ cells, these two miRNAs do not intervene in the process. However, when HIV infects the CD4^+^ T-cells, the resulting modulation of these two miRNAs in these cells likely sets off an otherwise virus independent or nonspecific role, which transforms into a dependent role in the hematopoietic CD34^+^ cells.

### Antiretrovirals for cytopenias–pros and cons

Antiretrovirals attenuate HIV replication and indirectly reduce different types of pancytopenias at random for a finite duration of the initial HIV/AIDS progression and subsequent containment using ART. However, in a chronic disease, the cytopenias eventually become symptomatic despite the use of antiretrovirals to reduce virus replication *in vivo* to the minimal and even virus levels designated as undetectable (<50 to <200 copies of HIV/mL of blood, or even less), depending on the different infected individuals. Hence, drug therapeutic candidates that intervene directly in the molecular mechanistic pathways of the onset of cytopenias are very necessary. It is also quite possible to be expected that the antiretrovirals modulate miRNA levels in the extracellular microenvironments by increasing or decreasing their secretion levels when the antiretroviral therapies as are known to attenuate the virus replication in SCID-hu Thy/Liv animals *in vivo* (Withers-Ward et al., 1997). We had previously reported that a synthetic isomer of biologically occurring sulfatide, 3′-O-galactosylceramide, rescues hematopoiesis and attenuates the virus replication more efficiently than treatment with the nucleoside analog reverse transcriptase inhibitor azidothymidine (AZT, Zidovudine) in these animals *in vivo* (Sundell et al., 2010). It is of interest whether the persistent and chronic HIV/AIDS disease, despite continued administration of antiretroviral therapies, disrupts the normal regulation of miRNA levels in the primary infection event in CD4^+^ thymocytes or in the secondary influence event in/on CD34^+^ HSPCs, which necessitates further studies on how HIV causes miRNA mediated dysregulation prior and subsequent to the miRNA–mRNA/HOX interactions (Figure 3). Horizontal intercellular communication (viz. involvement of exosomes) between the HIV-infected CD4^+^ thymocytes and this virus infection-resistant CD34^+^ HSPCs through miRNA transfer from the CD4^+^ to CD34^+^ cells requires continued investigation.

### Potential role of exosomes in the CD4^+^(miRNA)–CD34^+^(mRNA/HOX) interactions

The potential involvement of exosomes in the pathogenesis of HIV-induced hematopoietic inhibition through intercellular interactions involving microRNA target homeobox messenger RNA is of our imminent interest. Exosomes include miRNAs, long noncoding RNAs, mRNAs, proteins, and DNAs and contribute to the pathogenesis in viral infections and malignancies (Sasaki et al., 2019). It was reported that the immunosuppressive activity of exosomes from granulocytic myeloid-derived suppressor cells in a murine model delivered certain level of relief from immune bone marrow failure (Manley et al., 2023).

### Concluding hypotheses

Prolonged use of antiretroviral drugs in chronic HIV infections can cause symptomatic, resistant, or resilient and persistent pan/cytopenias and immune suppression in already immune-compromised virus-infected patients. HIV-1-induced hematopoietic inhibition occurs via dysregulated CD4^+^ T-cell-secreted miRNA-mediated alterations in the post-transcriptional regulation of the homeobox (HOX)-encoded transcription factors. Subsequently, the growth factor cytokines available to the lineage commitment and differentiation of the CD34^+^CD38^−^ HSPCs are disrupted through the specific signaling pathways as outlined, viz. MAPK (Figures 1, 3, 4). These events that occur initially through inter-cellular interactions and then transition into intra-cellular molecular changes that affect the CD34^+^ HSPCs’ fate determination cause abnormal hematopoiesis. We hypothesize that the virus-affected microRNAs target the MAPK signaling pathway via HOX genes in the regulation of CD34^+^ cell differentiation, leading to consequences on hematopoietic lineage stem cell fate (Nowicki et al., 2021). Further characterization of these mechanisms is needed to evaluate interventional miRNA therapies for sustained blood cell formation. Thus, these results and further proposed approaches have significant implications for the development of miRNAs as drug therapeutic candidates efficacious against specific cytopenias that occur in HIV/AIDS.

## Data Availability

The original contributions presented in the study are included in the article/Supplementary Material; further inquiries can be directed to the corresponding author.

## Abbreviations

AIDS, acquired immunodeficiency syndrome; Ang, angiopoietin; BFU-E, burstforming unit-erythroid; CFU-GM, colonyforming unit-granulocyte macrophage; CFU-MK, colonyforming unit-megakaryocyte; CD34, cluster of differentiation-34; *c-mpl*, cellular *proto-oncogene* of myeloproliferative leukemia; GM-CSF, granulocyte macrophage-colony stimulating factor; HSPCs, hematopoietic stem-progenitor cell(s); HOX, homeobox; HIV-1, human immunodeficiency virus type-1; IL-3/6, interleukin-3/6; PMG, promegapoietin; SCF, stem cell factor; SCID-hu Thy/Liv, severe combined immuno-deficient mouse transplanted with human fetal thymus and liver tissues; TPO, thrombopoietin; MAPK, mitogen-activated protein kinase-1/2 (MEK1/2)/extracellular signal-regulated kinase-1/2 (ERK1/2); mRNA, messenger RNA; miR/miRNA, microRNA; [Ca(i)^2+^], intracellular Ca^2+^.
